# Sex-Based Effects of Branched-Chain Amino Acids on Strength Training Performance and Body Composition

**DOI:** 10.3390/sports12100275

**Published:** 2024-10-11

**Authors:** Antonella Muscella, Maurizio Felline, Santo Marsigliante

**Affiliations:** Department of Biological and Environmental Science and Technologies (DiSTeBA), University of Salento, 73100 Lecce, Italysanto.marsigliante@unisalento.it (S.M.)

**Keywords:** branched-chain amino acid (BCAA), physical performance, delayed onset muscle soreness (DOMS), muscle recovery, fatigue perception

## Abstract

Background: Branched-chain amino acids (BCAAs) are widely studied for their effects on muscle recovery and performance. Aims: This study examined the effects of BCAA supplementation on anthropometric data, physical performance, delayed onset muscle soreness (DOMS), and fatigue in recreational weightlifters. Methods: The trial involved 100 participants (50 men and 50 women), randomized into BCAA and placebo groups. Subjects in the BCAA group took five daily capsules of 500 mg L-leucine, 250 mg L-isoleucine, and 250 mg L-valine for six months. A two-way ANOVA was used to analyze the main and interaction effects of sex and treatment. Results: Notable findings include significant improvements in muscle recovery, as indicated by reduced DOMS, particularly in women who showed a decrement of 18.1 ± 9.4 mm compared to 0.8 ± 1.2 mm in the placebo group of a horizontal 100 mm line. Fatigue perception was also significantly lower in the BCAA group, with women reporting a greater decrease (2.6 ± 1.5 scores) compared to the placebo group (0.6 ± 0.7 scores). Strength gains were prominent, especially in men, with a 10% increase in bench press maximum observed in the BCAA group. The interaction between sex and treatment was significant, suggesting sex-specific responses to BCAA supplementation. Conclusions: These results underscore the effectiveness of BCAA supplementation in enhancing muscle recovery, reducing fatigue, and improving strength. This study also highlights sex-specific responses, with women benefiting more in terms of DOMS and fatigue reduction, while men experienced greater strength gains, suggesting a need for tailored supplementation strategies.

## 1. Introduction

Physical exercise plays a fundamental role in the development of skeletal muscle, directly influencing energy expenditure and the use of macronutrients for energy purposes [1,2]. Certain essential amino acids, known as branched-chain amino acids (BCAAs), play a crucial role in improving body composition and sports performance and are essential for maintaining a healthy body, which is a body that maintains optimal muscle mass, low levels of fat mass, and balanced nutritional status [3,4,5]. Among the nine essential amino acids, three are branched chain (BCAAs): leucine, isoleucine, and valine [6], representing 40% of the mammalian amino acid requirement [7]. Within muscle proteins, they represent about one-third of skeletal muscle, while the amount in free form is marginal. Unlike other amino acids, BCAAs are metabolized exclusively in skeletal muscle because the BCAA amino-transferase enzyme is absent in the liver [8].

Furthermore, their catabolism is significantly favored by physical exercise [9]. The enzymes regulating BCAA catabolism and the entire catabolic pathway are located in mitochondria. The first reaction in catabolism is a transamination catalyzed by branched-chain amino-transferase. The second reaction is the irreversible oxidative decarboxylation catalyzed by the branched-chain α-ketoacid dehydrogenase complex (BCKDH), whose activity is strongly increased by physical exercise, particularly endurance exercise [9]; conversely, a low-protein diet or protein deficiency inactivates it [10]. Some studies have highlighted the relationship between two catabolic systems activated by physical exercise: fatty acid oxidation and BCAA oxidation [11]. Physical exercise promotes fatty acid oxidation, and BCKDH complex activation may be related to the increased fatty acid oxidation during physical activity [9]. Additionally, muscle protein synthesis is improved after exercise, justifying the increased requirement [12]. Finally, BCAA supplementation before and after exercise has beneficial effects by reducing exercise-induced muscle damage and promoting muscle protein synthesis, suggesting that BCAAs can be a useful supplement for physical exercise and sports in general [13]. As a matter of fact, BCAAs are one of the most popular sports supplements, despite ongoing controversies regarding their efficacy in sports nutrition [14]. These controversies arise from the fact that some studies have shown that BCAAs, particularly leucine, play a critical role in muscle protein synthesis [15,16], whilst other research suggests that the effect of BCAAs is limited without the presence of all essential amino acids [3]. Regarding the role of BCAAs in recovery, BCAAs have been shown in some studies to reduce muscle soreness after exercise and speed up recovery, especially before or after intense workouts [17,18]. However, despite these findings, some studies indicate that BCAA supplementation has no significant effect on recovery or that the benefits are marginal compared to other forms of supplementation like complete protein or other EAAs [19].

The aliphatic side chain of BCAAs, featuring a branch of a carbon atom attached to three or more carbon atoms, confers high hydrophobicity, making BCAAs particularly effective in maintaining the stability of folded proteins and performing functions for globular proteins [20,21]. Leucine, for example, is considered the most effective BCAA for stimulating muscle protein synthesis and muscle hypertrophy, acting as a “trigger” and turning on the synthesis of new muscle protein [22]. While the effectiveness of protein supplementation in muscle hypertrophy and performance is well documented, the role of BCAAs alone is less clear. Numerous studies have shown that BCAA supplementation may improve muscle hypertrophy and strength performance [23]. However, conflicting evidence exists regarding their efficacy compared to complete protein supplements that provide all essential amino acids [22,24].

Studies evaluating the impact of BCAAs on performance suggest that supplementation had insignificant effects on performance outcomes in cyclists and runners [25,26,27,28], volleyball players [29], and soccer players [30,31], although variation in supplementation protocols must be taken into account. However, findings on the efficacy of BCAA supplementation on body composition should be interpreted with caution. In several studies involving endurance athletes [32], recreational runners [26], and cyclists [27], comparable changes in body weight were observed in BCAA and control groups after 7 days of oral supplementation [27]. The effects of BCAA on lean tissue changes and fat mass [27,32] also appear to be negligible.

Recent studies have shown significant sex differences in BCAA metabolism and physiological responses to BCAA supplementation, although both sexes benefit from BCAAs. In particular, women may experience more pronounced reductions in delayed onset muscle soreness (DOMS) and perceived fatigue after exercise than men, suggesting that BCAAs may enhance recovery and reduce fatigue more effectively for women [19,33,34].

Men generally experience a more pronounced increase in muscle protein synthesis in response to BCAA supplementation than women. This enhanced response in men is partly due to higher levels of testosterone, which amplifies the muscle-building effects of BCAAs. Testosterone is known to stimulate muscle protein synthesis more effectively, thus contributing to greater gains in muscle mass [19]. On the other hand, estrogen may have a different regulatory effect on muscle protein metabolism than testosterone [35].

Additionally, Devries et al. [36] found that women require a higher percentage of leucine to effectively stimulate muscle protein synthesis, suggesting they may have lower sensitivity to leucine. Although Devries’s research focuses on older adults, the findings have broader implications for understanding leucine’s role across different populations, including potential sex differences in metabolic responses. As a result, men may benefit more from standard doses of BCAAs, whereas women may need higher leucine concentrations or different formulations to achieve comparable benefits.

This highlights the importance of considering sex-specific responses when designing supplementation protocols to maximize efficacy for both sexes.

Although BCAA supplementation has been widely demonstrated to have benefits in muscle recovery and performance enhancement, evidence of differences in BCAA metabolism and effects between men and women is limited; therefore, this study aimed to explore the impact of BCAA supplementation on muscle recovery, fatigue, body composition, and strength in a diverse group of weightlifters. By examining these factors in both male and female participants, this study seeks to provide insights into how BCAA supplementation can be optimized for different sexes, ultimately contributing to more effective and personalized sports nutrition strategies. Finally, we hypothesize that BCAA supplementation will lead to significant improvements in muscle recovery and strength performance compared to placebo, with distinct differences observed between male and female participants.

## 2. Materials and Methods

### 2.1. Participants

An a priori power analysis was used to estimate the sample size and ensure that the study had sufficient statistical power to detect significant differences between the two groups (BCAA supplementation vs. placebo) for the outcomes of interest, aiming to reduce Type I (false positive) and Type II (false negative) errors. The analysis was conducted using G*Power version 3.1.9.4 (Düsseldorf, Germany), based on the family of *t*-tests that are appropriate for comparing means of two independent groups (e.g., BCAA vs. placebo), using the following parameters: a predicted medium effect size of Cohen’s d = 0.5, a power of 80%, and a significance level (alpha) of 0.05. Having an appropriate sample size ensured that it was sufficiently powered to detect significant differences between the BCAA and placebo groups.

**Figure 1:**
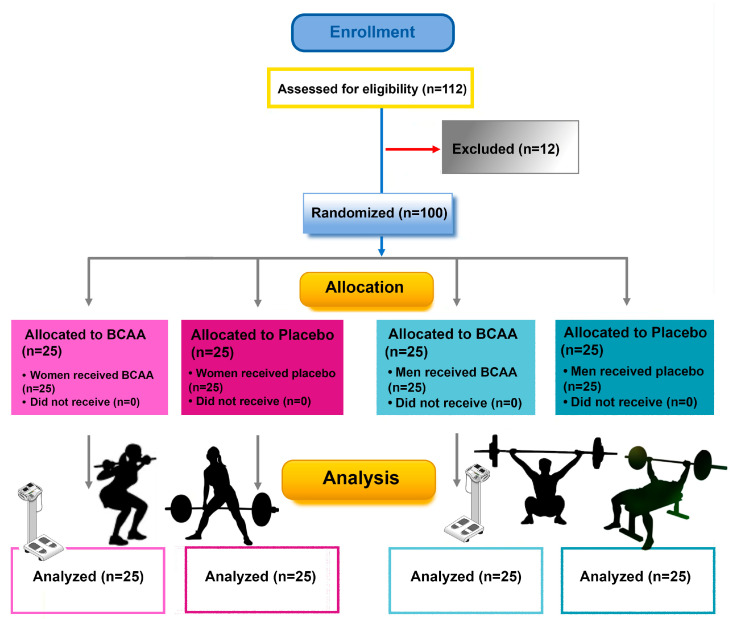
CONSORT flow diagram [37].

After a preliminary screening, the study involved 100 men and women recreational weightlifters aged between 20 and 48 years. For inclusion, participants met the following criteria: (a) at least three  years of powerlifting/weightlifting practice, with an average self-reported resistance training time of 4.5 ± 2.1 h per week; (b) they had to have a baseline fitness level that is similar across groups, as confirmed by initial fitness assessments; (c) adequate experience in performing the resistance exercises included in the program, to reduce the risk of injuries and ensure the reliability of the data collected; (d) they had to be in generally good health, with no chronic illnesses or conditions that might interfere with exercise or dietary supplementation; (e) availability to follow a standardized diet and to meticulously record daily food intake throughout the study, to minimize nutrition-related variables; (f) must be able to complete the prescribed strength training program and attend all scheduled assessments. We excluded those who developed medical conditions or injuries during the study that would affect their ability to safely engage in strength training or influence the study results (e.g., cardiovascular disease, severe musculoskeletal problems, metabolic disorders). We did not include individuals who were consuming creatine (within the past six months), were taking drugs that could affect muscle mass, fat mass, or general fitness (e.g., corticosteroids, anabolic steroids, some antidepressants), or who were involved in other interventions that could confound the results. We also excluded participants who were currently taking dietary supplements, as this could have influenced their response to the BCAA supplementation used in the study. We did not include individuals using treatments like cryotherapy or massage, and we excluded former or current smokers. Participants with recent or current involvement in a structured strength training program (e.g., within the past six months) were also excluded to avoid bias from pre-existing training adaptations. We also excluded participants with specific dietary restrictions or medical or psychological disorders that would affect their ability to adhere to the dietary or supplement requirements of the study. Participants consuming protein supplements (e.g., whey, casein) were asked to refrain from taking these during the study. In addition, all participants were required to abstain from exercise and alcohol consumption for 48 h before testing and throughout the entire testing period, as well as from caffeine for 12 h prior to each visit. Participants were made aware of all procedures’ risks and benefits, gave written consent, and completed health history, diet history, and physical activity questionnaires. During the study, no adverse effects were reported by any of the participants, and there were no dropouts throughout the entire study period. All participants adhered to the study protocols and completed the intervention as planned. In this study, a CONSORT flow diagram was applied (Figure 1).

### 2.2. Participant Retention and Compliance

Throughout the six-month intervention, no significant injuries, illnesses, or significant non-compliance events were observed, and all participants adhered to the intervention protocol as outlined without any withdrawals. We attribute this high retention rate to several factors:

- Addressing Minor Issues: While some participants experienced minor conflicts such as scheduling difficulties or mild discomfort, these issues were addressed promptly. Training schedules were temporarily adjusted to accommodate participants when necessary, ensuring compliance without affecting the study’s timeline.
- Experience of Participants: All participants had at least three years of prior powerlifting or weightlifting experience, with an average self-reported resistance training time of 4.5 ± 2.1 h per week. Their familiarity with training routines may have reduced the risk of injuries or overtraining, contributing to full participation.
- Study Timing: The study occurred between February and July, a period during which seasonal illnesses (such as influenza) were less prevalent, likely minimizing sickness-related absences. Respiratory illnesses, which are more common in the winter months and can cause temporary withdrawals, were rarely seen at this time of year.

### 2.3. Randomization

The randomization process for this study was performed to ensure that each participant had an equal chance of being assigned to either the BCAA supplementation or placebo group, thereby minimizing potential bias and confounding variables.

Participants were stratified by sex and baseline fitness levels before randomly assigning them to either the BCAA or placebo group. Then, randomization was performed with a computer-based random number generator resulting in two groups (BCAA supplementation and placebo) with equal numbers of men and women and balanced initial fitness levels. The randomization process also aimed to ensure that any potential confounding factors were evenly distributed between the two groups.

Randomization was verified by statistically controlling for the balance of anthropometric characteristics (e.g., age, height, weight, BMI, and baseline fitness levels) between the two groups. This verification ensured that the randomization process effectively balanced these characteristics between the groups (Table 1).

### 2.4. Training Program

The six-month resistance training program included 13 guided-motion resistance exercises, divided across three different training days. The training days were categorized into legs (leg press, leg curl, leg extensions, and standing calf raises), pushing exercises (seated military press, bench press, vertical bench press, chest fly, and seated machine triceps extensions), and pulling exercises (lat pulldown, seated wide-grip row, seated narrow low row, and seated biceps curl). Throughout the study, all participants trained 4 days/week. In the first 2 weeks, they performed 2 sets of 10–12 repetitions, at an initial intensity of approximately 70% of the pre-training 1RM. In the following weeks, the exercise intensity was increased to approximately 80–85% of 1RM to perform 3 sets of 6–10 repetitions. All training sessions were supervised by a study researcher (M.F.) to ensure correct technique and adherence to the prescribed exercise intensity. Participant compliance with the training program, in terms of attendance, exceeded 95% for everyone involved.

### 2.5. Fitness Assessments

One-repetition maximum (1 RM) was measured for each exercise before training and 3 d after the last training session to evaluate strength changes. Objective fitness assessments were performed by conducting a series of the following standardized “1 Repetition Maximum (1RM) test”: in order to determine the maximal weight that can be lifted once with proper form for exercises such as bench press and squat [38,39].

Both groups followed the same training program designed to improve physical performance and body composition.

Participant groups and blinding—Group 1 (BCAA supplementation) consisted of 50 subjects (25 men and 25 women) who supplemented their diet with BCAAs. Group 2 (placebo) consisted of 50 subjects (25 men and 25 women) that followed the same strength training program without BCAA supplementation.

Both groups received identical-looking capsules to maintain masking. Subjects in the BCAA group took five capsules per day for six months; each capsule contained 500 mg L-leucine, 250 mg L-isoleucine, and 250 mg L-valine. To ensure blinding, both groups received identical-looking capsules, with one group receiving BCAAs and the other a placebo. Furthermore, the experimenter analyzing the data was blinded to group assignments, which were marked with random letters to ensure unbiased analysis. We monitored supplementation compliance by participants returning empty containers of their supplement following the weeks of supplementation, and also by having them complete a weekly supplement compliance questionnaire.

Masking was critical to prevent potential bias from influencing participants’ perceptions or behavior during the study.

Strength training regimen was assessed following the Kraemer and Ratamess prescriptions [40]. In major detail, both groups followed a structured strength training program designed to improve physical performance and body composition. The training regimen was as follows: frequency: 3 sessions per week, on non-consecutive days; intensity: 10 min cardiovascular warm-up (e.g., treadmill, cycling); strength exercises: targeted major muscle groups using a combination of free weights and bodyweight exercises performed at 70–85% of the individual’s 1RM (one repetition maximum).

Sets and Repetitions—Core exercises: 3 sets of 8–12 repetitions for exercises such as squats, bench press, deadlifts, and pull-ups. Accessory exercises: 2–3 sets of 12–15 repetitions for exercises targeting smaller muscle groups (e.g., bicep curls, tricep extensions). Rest intervals: 60–90 s between sets to balance muscle recovery and workout intensity. Progression was scheduled as follows: load adjustment: weight increased by 5–10% every 4 weeks to ensure progressive overload. Exercises were periodically modified to prevent plateaus and maintain engagement.

Evaluation and Monitoring—For both groups, the following parameters were evaluated at the start and end of the study: body composition: lean mass, fat mass, and muscle mass measured using bioimpedance analysis (BIA). Performance metrics: changes in maximal strength (1RM) and overall performance. Subjective assessments: variations in the perception of delayed onset muscle soreness (DOMS) at 25 h post-training and subjective fatigue levels. Progress in muscle mass, fat mass, post-workout muscle soreness (DOMS), perception of fatigue, and strength performance was monitored throughout the study.

Body composition assessment by bioimpedance analysis (BIA)—BIA was used for assessing body composition, paying special attention to the hydration status of the participants. Body fat, muscle mass, and hydration status were estimated by using a AKERN Bia 101 Sport Edition (Pisa, Italy). This non-invasive technique measures the resistance (impedance) of body tissues to a small, safe electrical current, providing an estimate of various body compartments [41,42]. To ensure the accuracy of BIA, we took into account the constraints of the method. First, measurements are taken usually in the morning, to minimize hourly variability. Then, participants are often advised to hydrate adequately, as overhydration or dehydration can skew the results. Participants are typically asked to fast for 4–6 h and to avoid strenuous physical activity for at least 12–24 h before the measurement. Participants, who do not wear heavy clothing, metal accessories, or shoes, are asked to maintain a consistent posture to avoid discrepancies in the measurements.

The data were analyzed to observe trends in muscle mass, fat mass, and hydration status.

Diet Control—Diet was regulated as outlined by Arazi et al. [43]. Participants were instructed to maintain their usual diets throughout the study. Participants were provided with food diaries to record their daily food intake, which were collected and assessed periodically to monitor consistency and adherence to dietary instructions.

In cases where participants were found to have significantly deviated from the prescribed dietary intake, adjustments could be made, or those participants could be excluded from the final analysis. By implementing these controls and monitoring strategies, the study aimed to ensure that the observed effects were due to the BCAA supplementation and strength training program rather than confounding factors related to dietary intake. If significant differences were found, they were adjusted for in the statistical analyses to ensure that they did not confound the results.

Additionally, both written and verbal instructions were given for documenting the types and portion sizes of foods consumed 48 h prior to pre-test measurements. They were also told to replicate this diet 48 h before the post-test measurements. Analysis of the dietary data showed no significant differences between the groups in both pre- and post-test sessions. The protein intake ranged from 1.2 g/kg/day to 1.7 g/day. Carbohydrate intake ranged from 4.8 g/kg/day through to 6.2 g/kg/day; finally, the intake of fat ranged from 0.4 g/kg/day through to 1.6 g/kg/day.

### 2.6. Muscle Soreness Post-Exercise (DOMS) Assessment

DOMS was assessed at the beginning and end of the trial, following the 1-RM strength test. After an ascending warm-up (sets at 50%, 75%, and 90% of the estimated 1-RM), participants completed three 1-RM trials with 5 min recovery periods between each. DOMS was assessed using a visual analogue scale (VAS). The VAS was a horizontal 100 mm line, marked with 1–100 with the terminal descriptors no pain and severe pain. This method has been used previously as a non-invasive way to monitor changes in perceived pain following muscle damage [44,45,46,47]. Participants were requested to rate the sensation of discomfort 25 h after the exercise. Familiarization allows participants to become more familiar with the instrument, leading to more reliable self-reports, minimizing the possibility of misinterpretation, and ensuring that the data collected are accurate.

### 2.7. Perception of Fatigue Assessment

Fatigue was measured using the Rating of Fatigue (ROF) scale, an 11-point numerical scale ranging from zero to ten. This scale is effective and valid for assessing changes in fatigue across different settings [48]. The ROF scale is a straightforward, sensitive, and dependable method for monitoring fatigue perception during exercise and recovery. During the familiarization session, subjects received detailed instructions on how to rate their perceived fatigue. Twenty minutes after the initial and final training sessions, each participant rated their fatigue level from 0 (no fatigue at all) to 10 (complete fatigue and exhaustion).

### 2.8. Statistical Analysis

Statistical analyses were performed using using SPSS (version 24.0, IBM; Chicago, IL, USA). A balanced two-way ANOVA was used in order to assess the main and interaction effects of sex (male, female) and treatment (BCAA, placebo) on the dependent variables muscle recovery, strength performance, and fatigue. Post hoc tests (e.g., Tukey HSD) were conducted to explore significant interactions.

The paired-samples t-test within each group (the BCAA and the placebo groups) was used on paired data, pre- (T0) and post-treatment (T1) values for the same individuals, to see the changes over time. The independent-samples t-test was used to compare the mean changes in parameters of the two independent groups (BCAA group vs. placebo group) after 6 months.

A *p*-value of less than 0.05 was considered statistically significant, and results are reported as means ± standard deviations, with confidence intervals included where relevant.

## 3. Results

### 3.1. BCAA Effects on Body Composition

When considering the entire group (men and women, n = 100), the effects of BCAA supplementation were statistically significant for body fat mass and muscle mass (Table 2).

**Figure 2:**
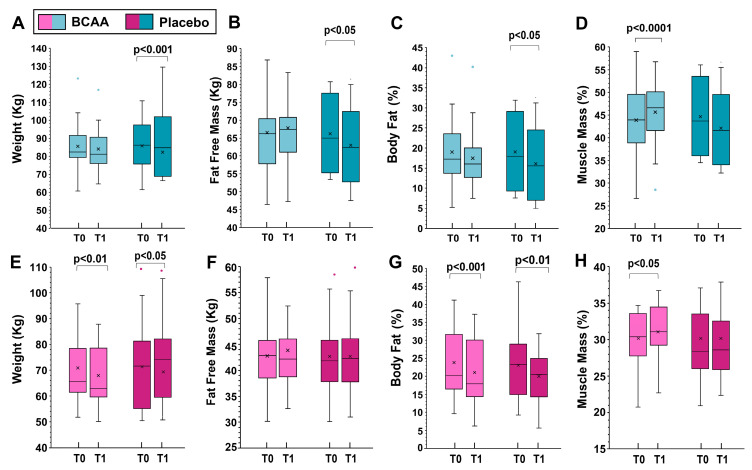
The effects of training alone (placebo group) and training with BCAA supplementation are shown for men (top, (**A**–**D**)) and women (bottom, (**E**–**H**)). T0 represents the start of the experiment (time zero) and T1 represents the end of the experiment (six months later). In this representation, the central box covers the middle 50% of the data values, between the upper and lower quartiles. The bars extend to the extremes, the central line represents the median, and the cross indicates the mean value. *p* values were obtained using a paired t-test comparing the values from the same subjects before and after the experiment.

When analyzing the sexes separately, statistically significant differences in women were observed in fat-free mass (increase of 1.1 ± 1.5 kg in the BCAA group and loss of −0.37 ± 0.5 kg in the placebo group, *p* < 0.001, by Tukey HSD) and muscle mass (increase of 2.1 ± 1.7 kg in the BCAA group and loss of 0.2 ± 2.7 kg in the placebo group, *p* < 0.001, by Tukey HSD), Figure 2.

For men, significant differences were noted in weight (increase of 0.18 ± 1.7 kg in the BCAA group and loss of 2.8 ± 1.8 kg in the placebo group, *p* < 0.001, by Tukey HSD), fat free mass (increase of 1.01 ± 0.5 kg in the BCAA group and loss of 0.53 ± 0.7 kg in the placebo group, *p* < 0.001, by Tukey HSD), and muscle mass (increase of 2.2 ± 1.0 kg in the BCAA group and decrease of 1.0 ± 1.8 kg in the placebo group, *p* < 0.001, by Tukey HSD). The data for men and women separately are shown in Figure 2.

### 3.2. BCAA Effects on Performance

When considering the entire group (men and women, n = 80), the effects of BCAA supplementation were statistically significant for all one-repetition maximum exercises (Table 3).

When analyzing the sexes separately, statistically significant differences in women were observed in 1-RM deadlift (increment of 9.4 ± 2.1 kg in the BCAA group and of 7.6 ± 1.7 kg in the placebo group, *p* = 0.009, by Tukey HSD).

For men, significant differences were noted in 1-RM squat (increment of 11.2 ± 3.5 kg in the BCAA group and 6.5 ± 0.8 kg in the placebo group, *p* < 0.001, by Tukey HSD), 1-RM on the bench press (increment of 9.5 ± 3.4 kg in the BCAA group and of 4.7 ± 1.8 kg in the placebo group, *p* < 0.001, by Tukey HSD), and for 1-RM deadlift (increment of 11.3 ± 2.2 kg in the BCAA group and of 8.1 ± 1.4 kg in the placebo group, *p* < 0.001, by Tukey HSD).

**Figure 3:**
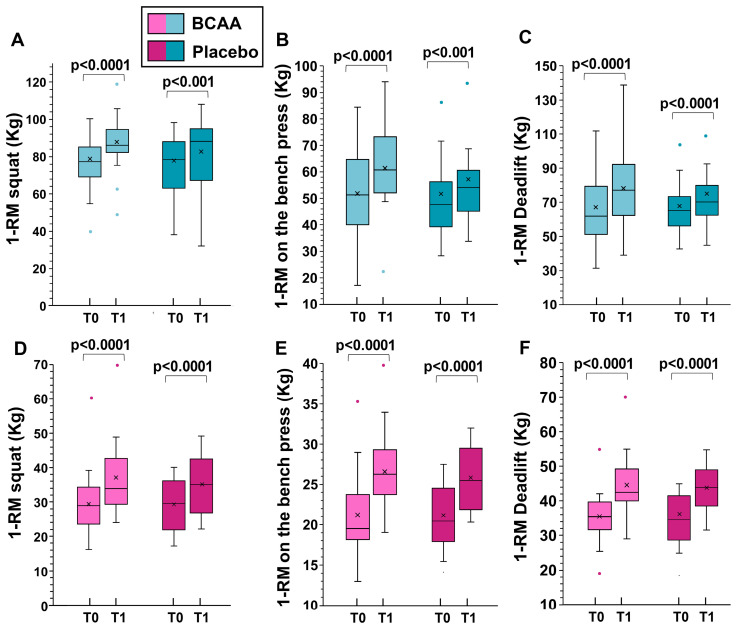
The effects of training alone (placebo group) and training with BCAA supplementation are shown for men (top, (**A**–**C**)) and women (bottom, (**D**–**F**)). T0 represents the start of the experiment (time zero) and T1 represents the end of the experiment (six months later). In this representation, the central box covers the middle 50% of the data values, between the upper and lower quartiles. The bars extend to the extremes, the central line represents the median, and the cross indicates the mean value. *p* values were obtained using a paired *t*-test comparing values from the same subjects before and after the experiment.

The data for men and women separately are shown in Figure 3.

### 3.3. BCAA Effects on DOMS and Perception of Fatigue

When analyzing the whole group (men and women, n = 80), BCAA supplementation had statistically significant effects on both DOMS and fatigue perception (Table 4).

**Figure 4:**
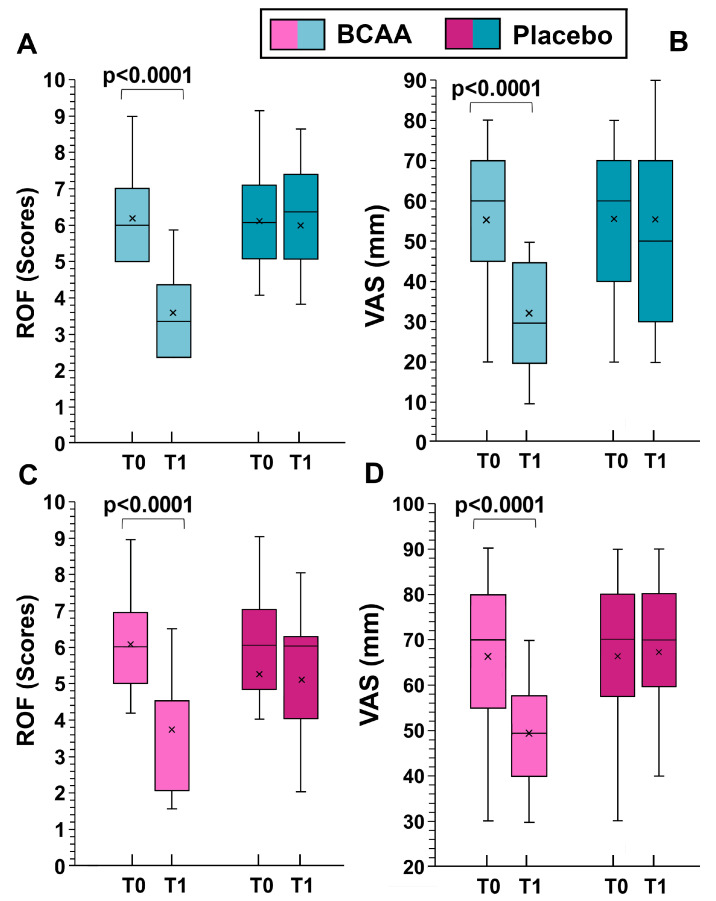
The effects of training alone (placebo group) and training with BCAA supplementation are shown for men (top, (**A**,**B**)) and women (bottom, (**C**,**D**)). T0 represents the start of the experiment (time zero) and T1 represents the end of the experiment (six months later). ROF stands for “rating of fatigue” and VAS stands for “visual analogue scale”. Probability values were obtained using a paired *t*-test comparing values from the same subjects before and after the experiment. In this representation, the central box covers the middle 50% of the data values, between the upper and lower quartiles. The bars extend to the extremes, the central line represents the median, and the cross indicates the mean value.

When analyzing the sexes separately, statistically significant differences in women were observed in DOMS (decrement of 18.1 ± 9.4 mm in the BCAA group and 0.8 ± 1.2 mm in the placebo group, *p* < 0.001 by Tukey HSD), and in fatigue perception (decrement of 2.6 ± 1.5 scores in the BCAA group and of 0.6 ± 0.7 scores in the placebo group, *p* < 0.001 by Tukey HSD), Figure 4.

For men, significant differences were noted in DOMS (decrement of 23.6 ± 10.4 mm in the BCAA group and of 0.1 ± 0.7 mm in the placebo group, *p* < 0.001 by Tukey HSD), and in fatigue perception (decrement of 3.0 ± 2.1 scores in the BCAA group and increment of 0.4 ± 0.4 scores in the placebo group, *p* < 0.001, by Tukey HSD). The data for men and women separately are shown in Figure 4.

## 4. Discussion

This study sought to investigate the overall effectiveness of BCAA supplementation, and the distinct effects between men and women. It revealed that BCAA supplementation effectively improved muscle recovery and reduced fatigue in both sexes, with pronounced gender-specific results: women experienced greater reductions in DOMS and fatigue, while men achieved more significant strength gains. These sex-specific responses to BCAA supplementation are noteworthy, as they suggest that supplementation strategies could be optimized by sex to maximize benefits. For example, women could prioritize BCAA supplementation to enhance recovery and alleviate fatigue, while men could leverage BCAAs to bolster strength gains.

Leucine, isoleucine, and valine are three essential branched-chain amino acids (BCAAs) that the body cannot produce and must be ingested exogenously [21]. BCAAs bypass liver metabolism and are oxidized in skeletal muscle [49]. Leucine activates the mammalian target of rapamycin-1 (mTOR), an anabolic signal that mediates muscle protein synthesis [21,50,51], which is related to adaptations in strength and hypertrophy [23,52]. Thus, BCAAs may benefit performance, recovery, and body composition [19]. Numerous studies have shown that BCAA supplementation reduces markers of muscle damage, such as creatine kinase (CK) and lactate dehydrogenase (LDH), post-exercise [53]. Furthermore, BCAAs have been shown to alleviate delayed onset muscle soreness (DOMS), aiding quicker recovery after strenuous exercise [19]. Despite the significant interest in BCAA supplementation, the number of studies focusing specifically on women is limited compared to those on men or mixed-sex groups [54,55,56]. Historically, much of the research on sports nutrition and supplementation, including BCAAs, has involved men due to controlled hormonal variability and greater participation in resistance training studies.

Here, we found that BCAA supplementation improves body composition and physical performance in both sexes. Men experience significant gains in muscle mass and strength, while women see more pronounced reductions in muscle soreness and fatigue, with less marked effects compared to men.

Our findings highlight the necessity for more targeted studies to understand the unique effects of BCAA supplementation in women, given the significant reductions in both DOMS and fatigue observed in women. For instance, studies have shown that women may experience greater reductions in perceived exertion and fatigue, potentially due to hormonal influences on amino acid metabolism [56,57]. Biological sex differences influence how BCAAs are processed and utilized by the body. Indeed, there are some important differences in amino acid metabolism between men and women starting from the hormonal influence: women experience increased protein oxidation, particularly during the luteal phase of the menstrual cycle when progesterone levels are elevated. This results in a higher requirement for amino acids during this phase compared to the follicular phase, with a specific increase in lysine requirements [58,59]. The progesterone surge enhances protein biosynthesis, reducing plasma amino acid levels due to processes such as endometrial thickening and increased protein utilization during exercise [59,60]. Consequently, women probably require more dietary protein than men due to this increased protein oxidation and biosynthesis. The suggested baseline for women is 1.6 g/kg/day, compared to a lower baseline of 1.2 g/kg/day for men [61,62]. Resistance training further increases these needs, especially for women who exercise. As a result, protein requirements differ between male and female athletes: men require approximately 1.7–2.2 g/kg/day, while female athletes require approximately 1.5–1.9 g/kg/day [63,64]. Furthermore, women tend to lose more fat mass and retain more lean mass than men during periods of caloric restriction [65,66]. The menstrual cycle also influences protein requirements, especially during the luteal phase, where increased protein oxidation increases the protein requirement for exercising women [59,60]. This phase is characterized by increased protein catabolism, requiring increased dietary intake to support muscle recovery and maintenance. Finally, women benefit from nutrient timing, with studies showing that protein consumption before and after exercise improves muscle protein synthesis, strength, and lean mass, particularly in trained women [67,68].

In men, BCAA supplementation is often associated with significant improvements in muscle mass and strength [52]. This effect is likely due to the higher baseline muscle mass and testosterone levels in men, which enhance the anabolic effects of BCAAs. In our study muscle mass increased significantly in both men and women, from 35.5 kg to 36.9 kg on average in men and with comparable gains observed in women. Research has demonstrated that men obtained greater results in muscle protein synthesis and lean body mass when supplementing with BCAAs, especially when combined with resistance training [4,5,57,69]. Also, in women, BCAA supplementation positively influences body composition by reducing body fat and increasing lean body mass, though these effects can be less pronounced compared to men and are often influenced by hormonal differences and baseline muscle mass [70]. Women may benefit more from the fat oxidation properties of BCAAs, potentially aiding in weight maintenance rather than significant weight loss [71]. Additionally, women have shown greater increases in plasma amino acids and fat-free mass during intense training compared to men, suggesting a different metabolic response to BCAA supplementation [72]. This might be related to lower testosterone levels, influencing how BCAAs are utilized in the body. For instance, Margolis and Pasiakos [72] found that women had greater increases in essential amino acids and fat-free mass during military training compared to men, highlighting sex-specific responses to BCAA supplementation.

BCAA supplementation affects athletic performance in both men and women, albeit with some differences in outcomes and underlying mechanisms.

Consistent with the increase in muscle mass, we demonstrated that BCAA supplementation led to a substantial increase in strength, with a 10% increase in 1-RM bench press compared to less than 5% in the control group, in men. However, statistically significant differences in performance in the 1-RM squat, the bench press, and the deadlift were also observed for women. In contrast, Wisnik et al. [73] found that BCAAs significantly improve psychomotor performance in men during high-intensity exercises but not in women, suggesting a greater capacity in men to oxidize BCAAs for energy.

In men, BCAA supplementation improves performance especially under stress and during high-intensity exercises, indicating a superior capacity to oxidize BCAAs for energy [56,73,74]. BCAAs improve endurance and reduce fatigue by modulating the tryptophan–serotonin pathway, which helps decrease perceived exertion during prolonged exercise [75]. This effect is particularly beneficial for activities requiring sustained effort, as BCAAs enhance performance by sparing glycogen and increasing fat utilization for energy [69]. Research by Blomstrand et al. [57] supports these findings by demonstrating that BCAA supplementation augments muscle protein synthesis, improving recovery. Moreover, BCAA supplementation improves exercise performance in resistance-trained individuals, emphasizing its role in reducing fatigue and enhancing recovery across sexes [76,77].

In women, BCAAs contribute to improved endurance and recovery and also significantly reduces muscle soreness and speeds up recovery times after intense exercise [19,56].

Overall, while BCAAs support athletic performance and recovery for both men and women, the extent of these benefits can vary, reflecting differences in hormonal and metabolic responses [19,57,69,75,78].

Consistent with our findings, previous studies showed that BCAA supplementation reduces muscle soreness from 24 to 96 h but not immediately after exercise-induced muscle damage [17,79,80,81].

Our study supports these findings, demonstrating that BCAA supplementation significantly decreases both DOMS and fatigue perception in both men and women. Specifically, the decrement in DOMS was 16.2 ± 13.9 mm for women and 2.3 ± 1.2 mm for men. Fatigue perception also showed significant reductions with decrements of 2.9 ± 1.7 scores for women and 3.2 ± 0.6 scores for men.

Although few and with limited participants, previous studies have also shown that in women, BCAA supplementation tends to have more pronounced effects on reducing perceived fatigue and DOMS compared to men [55]. This might be due to the influence of estrogen on amino acid metabolism, affecting how BCAAs are utilized during exercise [57].

Research indicates that women benefit more from BCAAs in reducing the perception of fatigue and soreness, especially after endurance exercise [82]. Furthermore, men exhibit a more pronounced reduction in muscle damage markers and substances related to fatigue, such as lactate and ammonia, compared to women, which might be attributed to greater muscle mass and more efficient BCAA absorption [76,82].

It is important to note that BCAA supplementation reduces markers of muscle damage but does not significantly affect the perception of central fatigue, suggesting that BCAAs may be more useful for physiological recovery than for psychological factors [53].

Some limitations must be acknowledged concerning this study. First, although the sample size of 100 subjects is reasonable, it may not fully represent the broader population. The findings may not generalize to individuals outside the age range of 20 to 48 years or to populations with different fitness levels.

Secondly, the study’s six months might be insufficient to observe long-term effects or adaptations to BCAA supplementation. Longer-term studies could provide more insight into the sustainability and long-term benefits of BCAA supplementation.

Other important factors might be that in this study while an effort was made to control variables, other factors such as participants’ adherence to the training program or other lifestyle factors were not monitored. These could impact the effectiveness of both the training program and the BCAA supplementation.

While the high retention rate and zero dropout over the six-month study period strengthen the reliability of our findings, this result is unusual for long-term interventions. Several factors, such as the timing of the study during a period of low seasonal illness, the experience level of participants, and the adjustments made for minor scheduling conflicts, likely contributed to the full retention.

However, it is important to acknowledge that this could be considered a limitation, as such a high retention rate may not be generalizable to other populations or settings. Studies involving less experienced participants or conducted over a different period may encounter higher dropout rates due to injuries, illnesses, or non-compliance. Future research should explore whether these factors influence retention in longer interventions or different populations.

## 5. Conclusions

This study examined the effects of BCAA supplementation on strength performance and body composition, with a focus on sex differences. The results highlight the potential of BCAAs to enhance athletic performance and muscle recovery in both men and women. However, the benefits were not uniform, revealing sex-specific responses. In women, BCAA supplementation was particularly effective in supporting recovery and reducing post-exercise muscle soreness. Incorporating BCAAs into their training regimens could, therefore help women minimize downtime and improve overall recovery. In contrast, in men, BCAA supplementation promoted muscle hypertrophy and improved strength performance. These sex-specific results are significant, as they not only confirm the efficacy of BCAAs in athletic performance but also highlight the need for tailored supplementation strategies that take into account the distinct physiological responses of men and women. By optimizing BCAA use based on gender, athletes can achieve more targeted and effective results. The study also raises important questions about the underlying biological mechanisms that drive these differences. Hormonal influences, variations in muscle fiber composition, and different metabolic pathways between men and women are potential areas of exploration that could further explain these sex-specific responses. Evaluating the long-term effects of BCAA supplementation in diverse populations is important to better understand how BCAAs can be used safely and effectively over extended periods. Future research should build on these findings by exploring how BCAAs can be integrated into broader nutritional and training strategies to support performance in both men and women.

## Figures and Tables

**Table 1:** Characteristics of participants.

		Women				Men		
	BCAA (n = 25)	95% CI	Placebo (n = 25)	95% CI	BCAA (n = 25)	95% CI	Placebo (n = 25)	95% CI
Age (years)	35.3 ± 11.5	30.8–39.8	34.3 ± 8.5	30.9–37.6	37.3 ± 11.5	32.7–41.8	36.8 ± 8.5	33.5–40.1
Height (cm)	165.0 ± 6.2	162.6–167.4	163.2 ± 7.3	160.3–166.0	173 ± 6.7	170.3–175.6	172.6 ± 6.9	169.9–175.3
Weight (kg)	71.3 ± 12.8	66.4–76.3	71.6 ± 13.4	66.4–76.9	85.6 ± 15.4	79.5–91.6	85.7 ± 16.8	79.1–92.3
BMI (kg/m^2^)	25.9 ± 4.5	24.1–27.7	26.1 ± 6.2	23.7–28.5	28.4 ± 4.1	26.8–30.0	29.0 ± 6.8	26.3–31.7
% Body fat	23.6 ± 8.2	20.4–26.8	23.0 ± 8.9	19.5–26.5	18.9 ± 4.9	17.0–20.9	19.1 ± 7.3	16.2–21.9
Free fat mass (kg)	42.7 ± 6.8	40.0–45.4	42.6 ± 6.4	40.1–45.1	66.6 ± 7.1	59.8–67.9	64.5 ± 12.4	59.6–69.3
Muscle mass (kg)	30.2 ± 3.2	28.9–31.4	29.6 ± 3.8	28.1–31.1	44.2 ± 6.4	41.6–46.7	44.65 ± 5.	41.6–46.7
Training years	3.9 ± 1.2	3.4–4.3	4.0 ± 1.3	3.6–4.6	4.1 ± 1.1	3.6–4.5	4.3 ± 1.3	3.8–4.7

Data are means ± S.D., BMI, body mass index. All data were compared using an unpaired *t*-test, and no significant differences (*p* > 0.05) were found among the groups.

**Table 2:** The mean changes anthropometric characteristics of the two independent groups.

Body Composition	Δ BCAA Group	Δ Placebo Group	Cases	*F*	*p*	η^2^
Weight	−2.06 ± 3.9	−3.3 ± 2.1	Treatment	8.645	0.004	0.052
			Sex	43.317	<0.001	0.261
Fat free mass	1.1 ± 1.2	−0.5 ± 2.3	Treatment * Sex	17.866	<0.001	0.108
			Treatment	62.246	<0.001	0.392
			Sex	0.499	0.482	0.003
			Treatment * Sex	0.046	0.830	0.00029
Fat mass	−2.3 ± 2.5	−2.7 ± 2.0	Treatment	1.130	0.290	0.011
			Sex	0.283	0.596	0.003
Muscle mass	2.2 ± 1.3	−0.6 ± 2.3	Treatment * Sex	3.106	0.081	0.031
			Treatment	53.970	<0.001	0.354
			Sex	0.798	0.374	0.005
			Treatment * Sex	1.538	0.218	0.010

*p* by two-way ANOVA.

**Table 3:** The mean changes in physical performances of the two independent groups.

Physical Performance	Δ (kg) BCAA Group	Δ (kg) Placebo Group	Cases	*F*	*p*	η^2^
1-RM squat	9.4 ± 3.3	6.4 ± 1.7	Treatment	54.738	<0.001	0.293
			Sex	20.587	<0.001	0.110
			Treatment * Sex	15.542	<0.001	0.083
1-RM on the bench press	7.6 ± 3.2	7.7 ± 1.3	Treatment	48.868	<0.001	0.260
			Sex	22.628	<0.001	0.120
			Treatment * Sex	20.790	<0.001	0.110
1-RM Deadlift	10.3 ± 2.4	7.8 ± 1.6	Treatment	41.643	<0.001	0.274
			Sex	11.185	0.001	0.074
			Treatment * Sex	3.274	0.074	0.022

*p* by two-way ANOVA.

**Table 4:** Mean changes in fatigue and DOMS of the two independent groups.

	Δ BCAA Group	Δ Placebo Group	Cases	*F*	*p*	η^2^
Fatigue	−4.4 ± 2.6	−0.6 ± 0.6	Treatment	134.840	<0.001	0.514
			Sex	18.216	<0.001	0.069
			Treatment * Sex	13.528	<0.001	0.052
DOMS	−20.0 ± 10.2	−0.5 ± 0.9	Treatment	170.238	<0.001	0.615
			Sex	6.263	0.014	0.023
			Treatment * Sex	4.379	0.039	0.016

## Data Availability

The data that supports the findings of this study are available on reasonable request from the corresponding author.
